# Osteoclast-derived small extracellular vesicle as novel liquid biopsy for assessing bone health in older adults

**DOI:** 10.1093/geroni/igaf122.4167

**Published:** 2025-12-31

**Authors:** Shalini Mishra, Yixin Su, Ashish Kumar, Sangeeta Singh, Kristen Beavers, Gagan Deep

**Affiliations:** Wake Forest University School of Medicine, Winston-Salem, North Carolina, United States; Wake Forest University School of Medicine, Winston-Salem, North Carolina, United States; Wake Forest University School of Medicine, Winston-Salem, North Carolina, United States; Wake Forest University School of Medicine, Winston-Salem, North Carolina, United States; Wake Forest University School of Medicine, Winston-Salem, North Carolina, United States; Wake Forest University School of Medicine, Winston-Salem, North Carolina, United States

## Abstract

Age-related bone loss is a highly prevalent condition that contributes substantially to morbidity, mortality, and healthcare costs worldwide. Dysregulated osteoclast activity characterizes aging bone; however, current diagnostic measurements lack specificity, sensitivity, and early detection ability. Small extracellular vesicles (sEV) derived from specific cell types offer a promising non-invasive liquid biopsy approach for assessing hard-to-access tissues, like bone. This proof-of-concept study aimed to isolate osteoclast-specific sEV (sEV-Osteoclast) from blood, laying the foundation for a sensitive, specific, and repeatable method to evaluate osteoclast physiology and molecular status of bone in clinical studies. Four osteoclast-specific surface markers (tartrate-resistant acid phosphatase (TRAP), receptor activator of nuclear factor kappa-Β (RANK), integrin alpha V (CD51) and dendritic cell-specific transmembrane protein (DCStamp)) were identified through extensive literature review and validated using the Human Protein Atlas and UniProt databases. Next, total sEV were isolated from human plasma samples (n = 11; age: 70.0±2.76 years) and characterized for size (99.65±2.49nm) and concentration (3.58E+11±3.13E+10particle/ml) by nanoparticle tracking analysis. Next, the percentage of sEVOsteoclast markers in the total sEV was ascertained by flow cytometry, revealing that 6.70±0.80% of plasma-total sEV were TRAP positive (+), 2.54±0.27% were RANK+, 7.36±0.64% were CD51+, and 3.63±0.27% were DCStamp+. As any specific sEV population can be reliably isolated from plasma if the percentage positivity is ≥ 2% in total sEV, these preliminary findings demonstrate feasibility of sEV-Osteoclast isolation from plasma. Future work aims to measure sEV-Osteoclast in a NIA-funded clinical trial of weight loss in older adults (NCT05764733), as a biomarker of early risk assessment and real-time intervention monitoring.

